# Supplementary material for: A Zero-Dimensional Organic Lead Bromide of (TPA)2PbBr4 Single Crystal with Bright Blue Emission
Source: Nanomaterials (Basel). 2022 Jun 28;12(13):2222. doi: 10.3390/nano12132222 (PMC9268179; doi:10.3390/nano12132222)
Supplement: Supplementary file 1 [file nanomaterials-12-02222-s001.zip › nanomaterials-1769764-supplementary.pdf]

Supplementary Materials

A Zero-Dimensional Organic Lead Bromide of $(TPA)_2PbBr_4$ Single Crystal with Bright Blue Emission

Ye Tian [1,2,†], Qilin Wei [2,†], Hui Peng [1,2,*], Zongmian Yu [2], Shangfei Yao [2], Bao Ke [2], Qiuyan Li [2] and Bingsuo Zou [2,*]

[1] Beijing Key Laboratory of Nanophotonics & Ultrafine Optoelectronic Systems, Beijing Institute of Technology, Beijing 100081, China; tianye080t@163.com

[2] Guangxi Key Lab of Processing for Nonferrous Metals and Featured Materials and Key Lab of New Processing Technology for Nonferrous Metals and Materials, Ministry of Education; School of Resources, Environments and Materials, Guangxi University, Nanning 530004, China; qlwei@st.gxu.edu.cn (Q.W.); yzmmaterials@163.com (Z.Y.); yaoshangfei@st.gxu.edu.cn (S.Y.); abnerkeb@163.com (B.K.); liqiuyan63@163.com (Q.L.)

* Correspondence: penghuimaterial@163.com (H.P.); zoubs@gxu.edu.cn (B.Z.)

† These authors contributed equally to this work.

Table S1. Crystal data and structure refinement for $(TPA)_2PbBr_4$ single crystal.

Empirical formula	$C_{24}H_{56}Br_4N_2Pb$
Formula weight	899.53
Temperature/K	297.7(5)
Crystal system	monoclinic
Space group	I2/a
a/Å	14.9268(6)
b/Å	14.5097(6)
c/Å	31.9309(11)
α/°	90
β/°	94.414(4)
γ/°	90
Volume/Å^3	6895.2(5)
Z	8
ρ_{calc}g/cm^3	1.733
μ/mm^{-1}	9.543
F(000)	3488.0
Crystal size/mm^3	0.21 × 0.19 × 0.16
Radiation	MoKα (λ = 0.71073)
2Θ range for data collection/°	3.084 to 61.798
Index ranges	$-15 \leq h \leq 21$, $-15 \leq k \leq 20$, $-42 \leq l \leq 45$
Reflections collected	26996
Independent reflections	9297 [R_{int} = 0.0417, R_{sigma} = 0.0697]
Data/restraints/parameters	9297/379/399
Goodness-of-fit on F^2	1.005
Final R indexes [I>=2σ (I)]	R_1 = 0.0478, wR_2 = 0.0960
Final R indexes [all data]	R_1 = 0.1323, wR_2 = 0.1138
Largest diff. peak/hole / e Å^{-3}	1.22/-1.23
Flack parameter	-0.07(7)

Table S2. Bond lengths for $(TPA)_2PbBr_4$ single crystal.

Atom	Atom	Length/Å	Atom	Atom	Length/Å
Pb1	Br1	3.0042(8)	N2	C16	1.510(6)
Pb1	Br2	2.8773(8)	N2	C16^{1}	1.511(6)
Pb1	Br3	2.7948(10)	C13	C14	1.500(8)
Pb1	Br4	2.7241(7)	C14	C15	1.502(8)
N1	C1	1.515(9)	C16	C17	1.515(8)
N1	C4	1.491(7)	C17	C18	1.518(9)
N1	C7	1.543(10)	N3	C19	1.620(13)
N1	C10	1.511(9)	N3	C19^{2}	1.620(13)
N1	C4A	1.518(10)	N3	C22	1.515(13)
N1	C1A	1.56(2)	N3	C22^{2}	1.515(13)
N1	C7A	1.48(3)	N3	C19A	1.432(16)
N1	C10A	1.53(3)	N3	C19A^{2}	1.432(16)
C1	C2	1.548(14)	N3	C22A^{2}	1.603(14)
C2	C3	1.479(11)	N3	C22A	1.603(14)
C3	C2A	1.52(3)	C19	C20	1.477(9)
C4	C5	1.526(8)	C20	C21	1.549(8)
C5	C6	1.538(8)	C21	C20A	1.536(9)
C6	C5A	1.526(9)	C22	C23	1.534(9)
C7	C8	1.476(15)	C23	C24	1.495(8)
C8	C9	1.525(11)	C24	C23A	1.550(9)
C9	C8A	1.63(3)	C4A	C5A	1.537(10)
C10	C11	1.492(12)	C1A	C2A	1.42(4)
C11	C12	1.518(11)	C7A	C8A	1.52(4)
C12	C11A	1.532(10)	C10A	C11A	1.534(10)
N2	C13^{1}	1.520(6)	C19A	C20A	1.530(9)
N2	C13	1.520(6)	C23A	C22A	1.477(9)

Table S3. Bond angles for $(TPA)_2PbBr_4$ single crystal.

Atom	Atom	Atom	Angle/°	Atom	Atom	Atom	Angle/°
Br2	Pb1	Br1	126.18(3)	C14	C13	N2	116.5(5)
Br3	Pb1	Br1	119.28(3)	C13	C14	C15	109.9(6)
Br3	Pb1	Br2	105.02(3)	N2	C16	C17	115.7(5)
Br4	Pb1	Br1	101.62(3)	C16	C17	C18	109.6(6)
Br4	Pb1	Br2	101.62(3)	C19	N3	$C19^{2}$	92.7(10)
Br4	Pb1	Br3	97.04(3)	$C22^{2}$	N3	$C19^{2}$	103.0(9)
C1	N1	C7	107.7(6)	C22	N3	C19	103.0(9)
C4	N1	C1	112.7(6)	$C22^{2}$	N3	C19	108.4(8)
C4	N1	C7	107.6(6)	C22	N3	$C19^{2}$	108.4(8)
C4	N1	C10	110.4(5)	$C22^{2}$	N3	C22	133.9(13)
C10	N1	C1	106.9(5)	$C22^{2}$	N3	$C22A^{2}$	32.3(5)
Br3	Pb1	Br1	119.28(3)	C13	C14	C15	109.9(6)
C10	N1	C7	111.5(6)	C22	N3	$C22A^{2}$	106.5(9)
C4A	N1	C1A	109.4(15)	$C19A^{2}$	N3	C19A	133.2(16)
C4A	N1	C10A	103.4(14)	$C19A^{2}$	N3	C22A	113.9(8)
C7A	N1	C4A	111.1(15)	C19A	N3	C22A	99.6(10)
C7A	N1	C1A	114.2(16)	$C22A^{2}$	N3	C22A	88.1(10)
C7A	N1	C10A	113.3(16)	C20	C19	N3	118.1(11)
C10A	N1	C1A	104.7(14)	C19	C20	C21	99.3(9)
N1	C1	C2	114.3(8)	N3	C22	C23	117.0(10)
C3	C2	C1	110.1(9)	C24	C23	C22	109.2(9)
N1	C4	C5	116.0(6)	N1	C4A	C5A	116.7(13)
C10	N1	C7	111.5(6)	C22	N3	$C22A^{2}$	106.5(9)
C4A	N1	C1A	109.4(15)	$C19A^{2}$	N3	C19A	133.2(16)
C4	C5	C6	107.7(6)	C6	C5A	C4A	105.2(10)
C8	C7	N1	116.5(8)	C2A	C1A	N1	112(2)
C7	C8	C9	108.2(8)	C1A	C2A	C3	114(2)
C11	C10	N1	116.6(7)	N1	C7A	C8A	115(2)
C10	C11	C12	109.5(8)	C7A	C8A	C9	104(2)
C13	N2	$C13^{1}$	107.0(6)	N1	C10A	C11A	111.0(16)
C16	N2	C13	111.0(3)	C12	C11A	C10A	108.5(16)
C16	N2	$C13^{1}$	110.8(3)	N3	C19A	C20A	119.8(12)
$C16^{1}$	N2	$C13^{1}$	111.0(3)	C19A	C20A	C21	111.2(11)
$C16^{1}$	N2	C13	110.8(3)	C22A	C23A	C24	102.7(8)
C16	N2	$C16^{1}$	106.3(6)	C23A	C22A	N3	115.5(11)
C4	C5	C6	107.7(6)	C6	C5A	C4A	105.2(10)
C8	C7	N1	116.5(8)	C2A	C1A	N1	112(2)

Table S4. Summary of emission peak, excitation peak , Stokes shift， FWHM, and PLQY of the low- dimensional metal halide with blue emission.

	PL (nm)	PLE (nm)	Stokes shift (nm) t	FWHM (nm)	PLQY	Reference
$[BAPrEDA]PbCl_6{\cdot}(H_2O)_2$	392 nm	300 nm	92 nm	73 nm	21.3%	[1]
$(C_{13}H_{19}N_4)_2PbBr_4$	460 nm	350 nm	110 nm	66 nm	~40%	[2]
$(C_9NH_{20})_7(PbCl_4)Pb_3Cl_{11}$	470 nm	348nm	112nm	84nm	83%	[3]
$(C_6N_2H_{16}Cl)_2SnCl_6$	450nm	375nm	75nm	125nm	8.1 %	[4]
$Cs_3Cu_2I_5$	445nm	290nm	155nm	110 nm	62.1%	[5]
Sb^{3+}-Doped $Cs_2NaInCl_6$	442nm	338nm	104nm	66nm	75.89%	[6]
$(C_5H_7N_2)_2ZnBr_4$	438nm	320nm	118nm	-	19.18%	[7]
$[(NH_4)_2]CuPbBr5$	441nm	288nm	153nm	107nm	32%	[8]
$[BAPrEDA]PbCl_6$	390nm	300nm	90nm	73nm	21.3%	[1]
Bi^{3+} Doped Cs_2HfCl_6	461nm	359nm	102nm	64nm	69.08%.	[9]
Bi^{3+} doped Cs_2SnCl_6	455nm	360nm	95nm	66nm	78.9%	[10]
$Cs_2AgSbCl_6$	409nm	325nm	84nm	-	31,33%	[11]
$Cs_3Bi_2Br_9$	410nm	330nm	80nm	48nm	19.4%	[12]
$FA_3Bi_2Br_9$	437nm	350nm	87nm	65nm	52%	[13]
$MA_3Bi_2Br_9$	423nm	356nm	67nm	62nm	12%	[14]
$(TPA)_2PbBr_4$	437nm	385nm	52m	50nm	12%	This work

Table S5. Simulation table of bond angles of ground state and excited state.

	Br5-Pb1-Br1	Br5-Pb1-Br13	Br5-Pb1-Br9	Br13-Pb1-Br1	Br13-Pb1-Br9	Br1-Pb1-Br9
ground-state	120.2419	103.6467	107.7008	99.5845	93.4944	124.9456
excited-state	121.1687	107.5212	104.7389	100.3904	91.0455	125.6806
distortion value	-0.9268	-3.8745	2.9619	-0.8059	2.4489	-0.735

Table S6. Simulation table of bond lengths of ground state and excited state.

	Pb1-Br1	Pb1-Br5	Pb1-Br13	Pb1-Br9
ground-state	3.01096	2.95914	2.78908	2.89284
excited-state	2.97842	2.9736	2.81145	2.91726
distortion value	0.03254	-0.01446	-0.02237	-0.02442

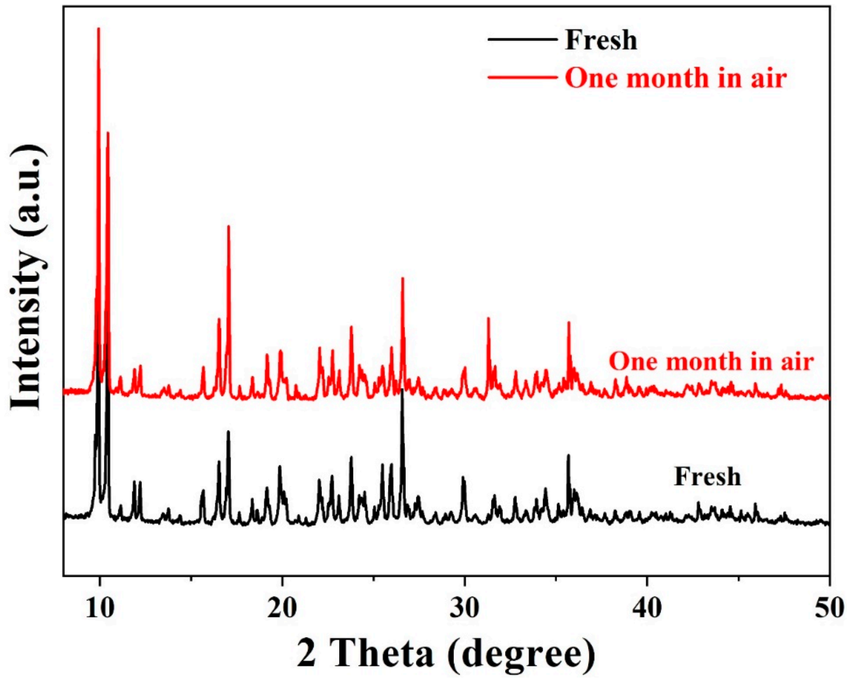

Figure S1. PXRD pattern result of $(TPA)_2PbBr_4$ powders after one month.

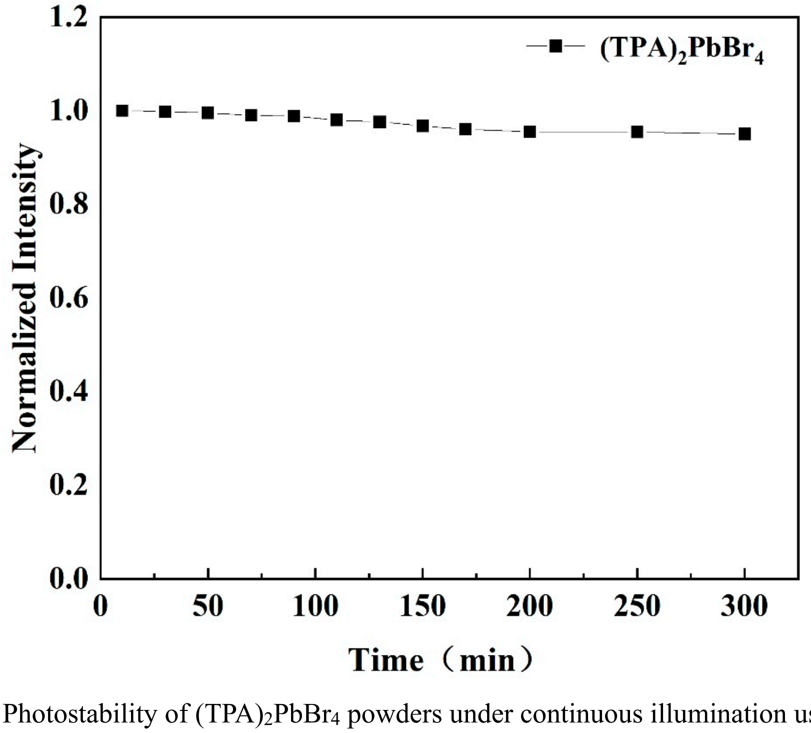

Figure S2. Photostability of $(TPA)_2PbBr_4$ powders under continuous illumination using a high power mercury lamp .

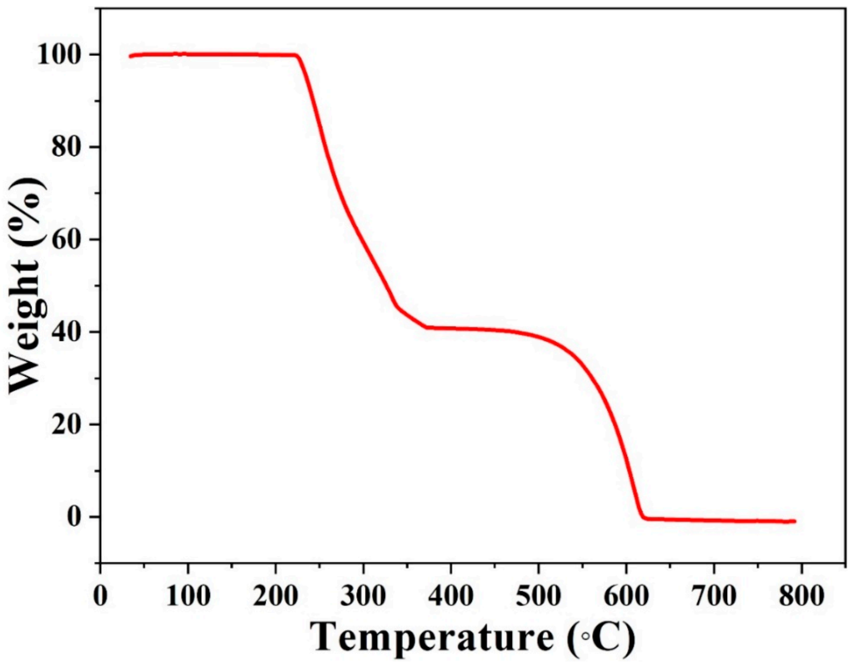

Figure S3. TG curve of $(TPA)_2PbBr_4$ powders.

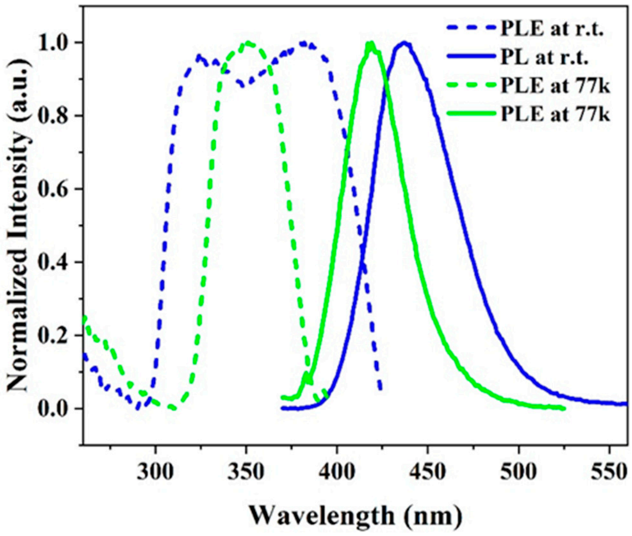

FigureS4. PL and PLE spectra at RT and 77 K of (TPA)2PbBr4 SCs crystals.

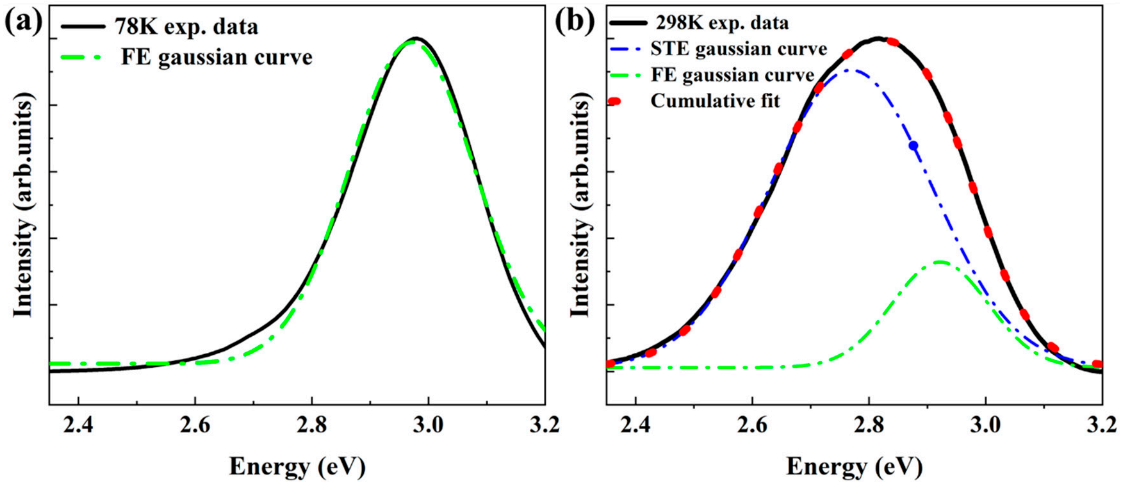

Figure S5. The PL spectra of $(TPA)_2PbBr_4$ SCs at (a) 78 and (b) 298 K were fitted by Gaussian curves.

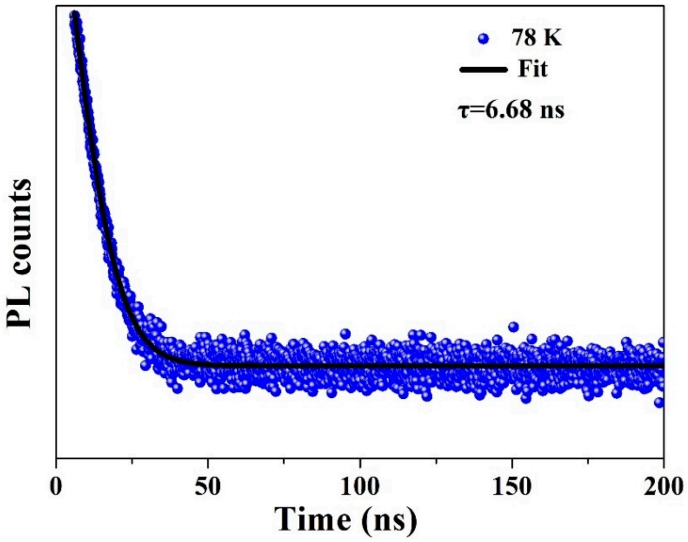

Figure S6. The decay lifetime curves of $(TPA)_2PbBr_4$ SCs at 98 K.

REFERENCES

(1) Sun, C.; Jiang, K.; Han, M.-F.; Liu, M.-J.; Lian, X.-K.; Jiang, Y.-X.; Shi, H.-S.; Yue, C.-Y.; Lei, X. A Zero-Dimensional Hybrid Lead Perovskite with Highly Efficient Blue-Violet Light Emission. *J. Mater. Chem. C* **2020**, *8*, 11890–11895.

(2) Lin, H.; Zhou, C.; Chaaban, M.; Xu, L.-J.; Zhou, Y.; Neu, J.; Worku, M.; Berkwits, E.; He, Q.; Lee, S.; Lin, X.; Siegrist, T.; Du, M.-H.; Ma, B. Bulk Assembly of Zero-Dimensional Organic Lead Bromide Hybrid with Efficient Blue Emission. *ACS Materials Lett.* **2019**, *1*, 594–598.

(3) Zhou, C. K.; Lin, H. R.; Worku, M.; Neu, J.; Zhou, Y.; Tian, Y.; Lee, S. J.; Djurovich, P.; Siegrist, T.; Ma, B. W. Blue Emitting Single Crystalline Assembly of Metal Halide Clusters. *J. Am. Chem. Soc.* **2018**, *140*, 13181–13184.

(4) S Song, G.; Li, M.; Yang, Y.; Liang, F.; Huang, Q.; Liu, X.; Gong, P.; Xia, Z.; Lin, Z. Lead-Free Tin(IV)-Based Organic-Inorganic Metal Halide Hybrids with Excellent Stability and Blue-Broadband Emission. J. Phys. Chem. Lett. 2020, 11, 1808–1813.

(5) Jun, T.; Sim, K.; Iimura, S.; Sasase, M.; Kamioka, H. Lead-Free Highly Efficient Blue-Emitting Cs3Cu2I5 with 0D Electronic Structure. *Adv. Mater.* **2018**, *30*, 1804547.

(6) Zeng, R.; Zhang, L.; Xue, Y.; Ke, B.; Zhao, Z.; Huang, D.; Wei, Q.; Zhou, W.; Zou, B. Highly Efficient Blue Emission from Self-Trapped Excitons in Stable Sb^{3+}-Doped $Cs_2NaInCl_6$ Double Perovskites. *J. Phys. Chem. Lett.* **2020**, *11*, 2053–2061.

(7) Yangui, A.; Roccanova, R.; McWhorter, T. M.; Wu, Y.; Du, M.-H.; Saparov, B. Hybrid Organic-Inorganic Halides $(C_5H_7N_2)_2MBr_4$ (M = Hg, Zn) with High Color Rendering Index and High-Efficiency White-Light Emission. *Chem. Mater.* **2019**, *31*, 2983–2991.

(8) Sun, C.; Guo, Y.-H.; Han, S.-S.; Li, J.-Z.; Jiang, K.; Dong, L.-F.; Liu, Q.-L.; Yue, C.-Y.; Lei, X.-W. Three-Dimensional Cuprous Lead Bromide Framework with Highly Efficient and Stable Blue Photo-luminescence Emission. *Angew. Chem., Int. Ed.* **2020**, *59*, 16465−16469.

(9) Liu, R.; Zhang, W.; Liu, W.; Li, G. Synthesis of a Bi^{3+}-Doped Cs_2HfCl_6 Double Perovskite with Highly Efficient Blue Light Emission at Room Temperature. *Inorg. Chem.* **2021**, *60*, 10451–10458.

(10) Tan, Z.; Li, J.; Zhang, C.; Li, Z.; Hu, Q.; Xiao, Z.; Kamiya, T.; Hosono, H.; Niu, G.; Lifshitz, E.; Cheng, Y.; Tang, J. Highly Efficient Blue-Emitting Bi-Doped Cs_2SnCl_6 Perovskite Variant: Photoluminescence Induced by Impurity Doping. *Adv. Funct. Mater.* **2018**, *28*, 1801131.

(11) Lv, K.; Qi, S.; Liu, G.; Lou, Y.; Chen, J.; Zhao, Y. Lead-free silver-antimony halide double perovskite quantum dots with superior blue photoluminescence. *Chem. Commun.* **2019,** *55*, 14741−14744.

(12) Leng, M.; Yang, Y.; Zeng, K.; Chen, Z.; Tan, Z.; Li, S.; Li, J.; Xu, B.; Li, D.; Hautzinger, M. P.; Fu, Y.; Zhai, T.; Xu, L.; Niu, G.; Jin, S.; Tang, J. All-Inorganic Bismuth-Based Perovskite Quantum Dots with Bright Blue Photoluminescence and Excellent Stability. *Adv. Funct. Mater.* **2018**, *28*, 1704446.

(13) Shen, Y.; Yin, J.; Cai, B.; Wang, Z.; Dong, Y.; Xu, X.; Zeng, H. Lead-free, stable, high-efficiency (52%) blue luminescent $FA_3Bi_2Br_9$ perovskite quantum dots. *Nanoscale Horiz.*

2020, *5*, 580−585.

(14) Leng, M.; Chen, Z.; Yang, Y.; Li, Z.; Zeng, K.; Li, K.; Niu, G.; He, Y.; Zhou, Q.; Tang, J. Lead-Free, Blue Emitting Bismuth Halide Perovskite Quantum Dots. *Angew. Chem. Int. Ed.* **2016**, *55*, 15012−15016.
